# On learning in the clinical environment

**DOI:** 10.1007/s40037-018-0441-x

**Published:** 2018-07-10

**Authors:** Matilda Liljedahl

**Affiliations:** 1 Department of Learning, Informatics, Management and Ethics, Karolinska Institutet, Stockholm, Sweden; 2 Primary Health Care Unit, Institute of Medicine, The Sahlgrenska Academy, University of Gothenburg, Gothenburg, Sweden

**Keywords:** Clinical education, Medical education, Socio-cultural perspectives, Workplace learning

## Abstract

**Introduction:**

The clinical environment has been increasingly acknowledged as an important setting for learning within healthcare professional education. In particular, researchers have highlighted the need to advance the knowledge on the social nature of learning in the workplace setting. The aim of the thesis was to explore workplace learning among undergraduate medical and nursing students.

**Method:**

The thesis adopted a socio-cultural perspective on learning and employed a qualitative approach embedded in an interpretative tradition of inquiry. Four consecutive studies were included in the thesis, the first one designed according to qualitative description whereas the other three had an ethnographic approach. Data were collected through individual interviews and field observations. Content analysis and thematic analysis were employed.

**Results:**

For the medical students, workplace learning entailed access to a variety of activities in the role of a marginal member of the healthcare team. Medical students demonstrated an adaptive approach to workplace learning. For the nursing students, workplace learning involved being entrusted to hold responsibility for patient care and the need to negotiate their basic values with those of the workplaces. Nursing students showed a hesitant approach to workplace learning.

**Discussion:**

Workplace learning was built upon varying theoretical perspectives of learning in the medical and nursing contexts respectively. The main message in the thesis argued for an upgrading of students as a powerful and active stakeholder in workplace learning, so as not to view students as passive consumers of clinical education.

## Introduction

Learning in the clinical environment has been extensively researched and established the clinical setting as a powerful environment for learning [1]. Nevertheless, students continually report on challenges in the workplace environment, such as the supervisor relationship, clerkship fatigue and the experience of being a guest at the clinical ward [2, 3]. In medical education, there has been a lack of attention on the social nature of learning [4], and researchers argue that such a perspective might shed light on under-recognized features of the clinical environment [5]. To address this gap in the literature, this thesis therefore intended to explore workplace learning of undergraduate students from a socio-cultural perspective. Workplace learning refers to learning situated in a setting originally and primarily designed for practice, that is, ‘work’ [6]. The socio-cultural concept of communities of practice [7] has previously been proven useful as a theoretical framework in medical education research. More novel theories include workplace participatory practices as outlined by Billett [8] and the framework of teaching and learning regimes [9], both of which can assist in advancing our understanding of clinical settings as learning environments for students. Workplace participatory practice views workplace learning as a relational interdependence between the affordances of a workplace (how opportunities and invitations to participate are offered) and individual engagement (how individuals elect to engage in the workplace) [8]. A teaching and learning regime is understood to be a set of implicit assumptions, rules and practices guiding the way in which teachers and students interact in relation to teaching and learning [9]. According to the framework of teaching and learning regimes, there can, for example, be dos and don’ts regarding how students are supposed to learn in a workplace, which is understood to be a manifestation of a current regime.

The overall aim of the thesis was to explore workplace learning among undergraduate medical and nursing students. Specifically, the thesis aimed to explore students’ experiences of the clinical learning environment (Study I), to explore the interdependence between workplace affordances and individual engagement in clinical learning environments of medical students (Study II) and nursing students (Study III) and, finally, to explore practices of workplace learning in the medical and nursing contexts (Study IV).

## Methods

The thesis was conducted within the interpretative tradition meaning that a relative worldview was adopted. Hence, it is acknowledged that several different realities can co-exist and that no study can fully give credit to all possible explanations of a phenomenon. In line with the theoretical framework adopted and the nature of the research questions, a qualitative approach was chosen as the overall method in the thesis. The studies were all set in large publicly funded hospitals in Stockholm, Sweden. In Sweden, the clinical phase of the 5.5-year medical program combines theoretical activities such as lectures and seminars with clinical rotations that last between 2 h to 21 days. The 3‑year nursing programs combine theoretical courses with clinical placements of approximately 2–8 weeks. Study I had a qualitative description design and included 15 semi-structured interviews with medical and nursing students which were subject to qualitative content analysis. Studies II–IV adopted an ethnographic approach and included 135 h of observational data and 16 follow-up interviews from three clinical sites, which were analyzed thematically. In studies II and III, data were analyzed inductively, whereas in Study IV data were analyzed deductively with a teaching and learning regime framework. Issues of trustworthiness were addressed in several ways. For instance, investigator and data triangulation was used as a way to enhance richness in data and analysis.

## Results

Study I [10] demonstrated how medical students accepted the placement as it was and they reported on not taking any actions if, for example, a placement did not work according to their expectations. Medical students experienced that they needed to promote themselves for supervisors to engage with them and they emphasized how they learned by observing supervisors’ practice. Nursing students on the other hand seemed to have high expectations of placements and talked of themselves as responsible for their learning. Nursing students did not want to be a ‘tail’ of the supervisor, and reported on how they strived to extricate themselves from the supervisor. They highly valued opportunities to be independent in the care of patients.

Study II [11] described how medical students were given access to activities in the clinical workplace in the role of a marginal player in the healthcare team where they were welcomed but not needed in the practice of healthcare. Workplaces expected clinical rotations to enable students to gain various experiences. Students appeared attentive to the role they were given and strived to fit into the workplace without disturbing healthcare practice. They did not necessarily engage in the afforded activities but could instead estimate the value of an activity and consequently, they were selective in terms of what they engaged in. Students were not bothered by the unscripted nature of learning activities but rather approached the complexity of rotations in an easy-going manner.

Study III [12] revealed how nursing students were entrusted to provide care for patients as they were offered membership in the clinical workplace. The membership offered was conditional in that students were expected to align with professional norms. Students appeared aspirational in their efforts to fill in the role they were offered and strived to handle the responsibility of patient care. Doing so, they seemed overwhelmed and responsibility was thus an energy-intensive assignment. Further, nursing students were exposed to a pragmatic reality of healthcare where they could strongly disagree with the workplace regarding views of patient care. Nursing students thus needed to challenge their own basic values and demonstrated a hesitance to adjust to the workplace culture.

Study IV identified four regimes of teaching and learning in the clinical environment. In the medical contexts, the two identified regimes both held an individual approach to learning but differed with regard to what was supposed to be learnt. In the *regime of reproducing practice *the most important things for students to learn were regularly emphasized whereas in the *regime of engagement in professional development* there was instead a focus on students’ conceptual understanding. In the nursing contexts, the two regimes both had a relational approach to learning but differed in how the student and supervisor interacted. In the *regime of participation in a partnership *the student and supervisor formed a close and trusting relationship. By contrast, in the *regime of membership in a stipulated community*, the student-supervisor relationship held a more formal character.

## Discussion

The thesis indicated that there are fundamental variations regarding how workplace learning is arranged and practised among medical and nursing students. These variations seemed to have their base in the underpinnings of learning in each context where workplace learning among medical students was characterized by an individual view on learning, meaning that medical students to some extent were left on their own in terms of learning; whereas workplace learning among nursing students instead was characterized by a relational view on learning, meaning that nursing students needed to succeed with workplace relationships to get access to learning.

Workplace learning among medical students was arranged to enable activities and experiences which would expose students to yet unexplored areas of clinical practice. This exposure to a variety of clinical practices seemed to be based upon the following understanding: *the greater the exposure to clinical practice students receive—the greater the students’ opportunities for learning will be*, a well-known assumption in rotation-based clinical education [13]. As students were afforded a marginal status, the invitational ability of workplaces can be considered as low and likewise, as students deselected available activities, they demonstrated a low degree of engagement. Workplace learning in the medical context was therefore characterized by a *bilateral detachment* where neither the workplace nor the students managed to engage enough for learning opportunities of high quality to be created.

Workplace learning among nursing students included the involvement of nursing students in practice as the workplace trusted them to care for patients. The establishment of relationships and membership in the workplace community were in focus; *the closer the relationship between the student and the workplace—the greater the students’ opportunities for learning will be*, which has also been recognized in previous research [14]. Here, however, students demonstrated a hesitance to align to workplace culture. Workplace learning in the nursing context was characterized by *dilemmas regarding loyalties* as students needed to choose to align to the workplace or follow their own beliefs.

Three theoretical frameworks were employed in this thesis. While communities of practice [7] was successful in emphasizing meaning and identity as crucial aspects of workplace learning, the involuntary nature of clinical education does not fit well into this framework. Additionally, as communities of practice is known as an informal and relational theory, it might underestimate the impact of hierarchies and power. Workplace participatory practices [8] was instead identified as a promising workplace learning theory as it acknowledges the bidirectional nature of workplace learning. The weaknesses here related to whether or not students are intended to be complete participants of workplaces and if students have the freedom to elect *not* to engage in clinical workplaces. Teaching and learning regimes [9] proved assistive in highlighting how power dynamics guide how workplace learning is put into practice. Its teacher-oriented nature and its development outside clinical environments are part of its limitations. I would argue for workplace participatory practices to replace communities of practice as the prominent workplace learning theory in medical education, not least as it acknowledges the contribution of individuals’ agency more successfully than communities of practice.

At the time this research was performed, I was a medical student or newly graduated medical doctor. The student role enabled power imbalances between the researcher and participants to be mitigated and I was also familiar with the context. However, it was, especially in the beginning, sometimes challenging to distinguish my own role as a student from that of a researcher. The thesis was, as much qualitative inquiry, limited to its context and restricted amount of data. By contrast, a significant effort was put into thorough analysis and theoretical anchoring of interpretations.

To conclude, the thesis demonstrated workplace learning to be built upon varying perspectives on learning in the medical and nursing context respectively. The main message in this thesis argues for an upgrading of students as a powerful and active stakeholder in workplace learning, so as not to view students as passive consumers of clinical education.

## Piece of advice

I started my PhD when I was still a medical student and brought all my own experiences as a medical student with me into the research. Exploring them from a scientific perspective opened doors for me into new ways of seeing and viewing the world. The most important piece of advice I can offer is to engage in something of your own interest and an idea you believe in. That will make your PhD journey so much more enjoyable.

## University information

The PhD thesis was defended at Karolinska Institutet in Stockholm, Sweden on 28 October 2016. Matilda Liljedahl was supervised by Dr Klara Bolander Laksov (main supervisor), Dr Erik Björck and Professor Sari Ponzer (co-supervisors). The full text of the thesis can be retrieved from: https://openarchive.ki.se/xmlui/handle/10616/45259.
